# Direct Access to Physical Therapy: Should Italy Move Forward?

**DOI:** 10.3390/ijerph19010555

**Published:** 2022-01-04

**Authors:** Filippo Maselli, Leonardo Piano, Simone Cecchetto, Lorenzo Storari, Giacomo Rossettini, Firas Mourad

**Affiliations:** 1 Department of Neurosciences, Rehabilitation, Ophthalmology, Genetic and Maternal Infantile Sciences (DINOGMI), Campus of Savona, University of Genova, 17100 Savona, Italy; 4313618@studenti.unige.it (F.M.); lorenzo.storari93@gmail.com (L.S.); 2 Sovrintendenza Sanitaria Regionale Puglia INAIL, 70126 Bari, Italy; 3 Fondazione dei Santi Lorenzo e Teobaldo, 12050 Rodello, Italy; l.piano@hotmail.it; 4 Direction of Health Professions, APSS, 38014 Trento, Italy; simone.cek@gmail.com; 5 School of Physiotherapy, University of Verona, 37129 Verona, Italy; giacomo.rossettini@gmail.com; 6 Department of Physiotherapy, LUNEX International University of Health, Exercise and Sports, 4671 Luxembourg, Luxembourg; 7 Luxembourg Health & Sport Sciences Research Institute A.s.b.l., Avenue du Parc des Sports 50, 4671 Luxembourg, Luxembourg

**Keywords:** direct access, physical therapy, cost-effectiveness, scope of practice, orthopedic manipulative physical therapy

## Abstract

Direct access to physical therapy (DAPT) is the patient’s ability to self-refer to a physical therapist, without previous consultation from any other professional. This model of care has been implemented in many healthcare systems since it has demonstrated better outcomes than traditional models of care. The model of DAPT mainly focuses on the management of musculoskeletal disorders, with a huge epidemiological burden and worldwide healthcare systems workload. Among the healthcare professionals, physical therapists are one of the most accessed for managing pain and disability related to musculoskeletal disorders. Additionally, the most updated guidelines recommend DAPT as a first-line treatment because of its cost-effectiveness, safety, and patients’ satisfaction compared to other interventions. DAPT was also adopted to efficiently face the diffuse crisis of the declining number of general practitioners, reducing their caseload by directly managing patients’ musculoskeletal disorders traditionally seen by general practitioners. World Physiotherapy organization also advocates DAPT as a new approach, with physical therapy in a primary care pathway to better control healthcare expenses. Thus, it is unclear why the Italian institutions have decided to recognize new professions instead of focusing on the growth of physical therapy, a long-established and autonomous health profession. Furthermore, it is unclear why DAPT is still not fully recognized, considering the historical context and its evidence. The future is now: although still preliminary, the evidence supporting DAPT is promising. Hard skills, academic paths, scientific evidence, and the legislature argue that this paradigm shift should occur in Italy.

## 1. Introduction

Direct access to physical therapy (DAPT) is the patient’s ability to self-refer to a physical therapist without previous consultation from any other professional (e.g., physicians or nurses) [1]. The World Physiotherapy organization strongly advocates this model of care, promoting a change in the mindset of government and the consumers about how DAPT can benefit healthcare systems and society [2]. Many healthcare systems (e.g., United States, United Kingdom, Australia) have implemented this model of care since it has demonstrated better clinical outcomes. Moreover, DAPT has reported a lower economic burden [3,4]—either for direct (e.g., number of visits, imaging) and indirect costs (e.g., lost workdays)—compared to traditional models of care (e.g., physician-centered paradigm) (for further details, refer to Table 1) [4,5,6,7]. The model of DAPT was mainly implemented to manage musculoskeletal disorders (MSDs) [8], a group of clinical conditions (e.g., low back pain, neck pain, shoulder pain) with a huge epidemiological burden and worldwide healthcare systems workload [9]. The Global Burden of Disease (GBD) study ranked MSDs—with low back pain and neck pain within the first five causes—within the top 20 leading causes of disability among the worldwide population [9]; focusing on the work-age population, MSDs become two of the ten leading causes for disability, with increased healthcare utilization and socioeconomic burden.

## 2. Discussion

Among healthcare professionals, physical therapists are one of the most accessed for managing pain and disability related to MSDs [10]. Additionally, it is recommended by the most updated guidelines as a first-line treatment because of its cost-effectiveness, safety, and patients’ satisfaction compared to other interventions [11,12].

Emerging evidence from recent studies emphasizes the potential role of DAPT in reducing costs associated with the care pathway: fewer visits, fewer exams, and a more active approach that may allow patients with MSD to achieve an earlier and better functional recovery [4,12]. Moreover, two recent randomized controlled trials have reported DAPT as an effective strategy even for patients with acute musculoskeletal pain in the emergency department [13] and a quick and safe adjunct to usual general practitioner-led primary care [14].

DAPT was also adopted to efficiently face the diffuse crisis of the declining number of general practitioners, reducing their caseload by directly managing those MSD patients traditionally seen by general practitioners [15]. This novel approach, with physical therapy in a primary care pathway, has been shown to reduce general practitioners’ workload and avoid unnecessary secondary care referrals (e.g., significant reduction of inappropriate referrals to orthopedics) [15]. Accordingly, with the goal to meet the needs caused by the pandemic, France also will fully implement the DAPT beginning in 2022 [16].

The extended scope physical therapist as primary care provider has also developed within Emergency Departments (ED) over the last years [17,18]. ED physical therapists have become a key resource within ED in certain countries in response to overcrowding, positively impacting waiting times, treatment times, length of stay, patients’ satisfaction, and costs by increasing the appropriateness of admission and diagnostic imaging [19,20].

These promising results of DAPT increased the interest in the topic leading to several novel publications [8,9,10,11,12,13,14,15]. Ohja et al. were the first to provide preliminary evidence that DAPT was associated with better outcomes and fewer costs than a referred pathway [21]. In 2017, a narrative review by Piano et al. reported that DAPT was associated with a higher patient’s satisfaction, lesser cost, and better or equal clinical outcomes for the management of MSD, when compared to other models of care (e.g., medical referral to physical therapy or standalone medical management) [22]. Although a retrospective study of 50,799 cases observed that DAPT does not result in a greater risk of adverse events than a referred pathway [23], there are still no firm conclusions regarding the safety of DAPT since few studies primarily focused on the adverse events. Additionally, Piscitelli et al. found similar results for the risk of adverse events in the whole physical therapy practice (e.g., not exclusively related to MSD), in addition to a reduction of direct and indirect costs (e.g., number of visits, number of X-ray referrals, medication intake, and working days lost) [24]. However, there is still no firm conclusion regarding the safety of DAPT since few studies primarily focused on adverse events [24]. Recently, Demont et al. found weak to moderate quality of evidence supporting DAPT as an effective intervention on disability, quality of life, and healthcare costs, but no difference for pain compared to physician-led management [6]. Physical therapy has positively impacted the healthcare system’s efficiency, reducing healthcare utilization (e.g., less imaging, medication, and secondary consultation) [6]. Moreover, physical therapy decreases general practitioners’ caseload and the socioeconomic burden of MSD (e.g., work absenteeism, sick leave), being more cost-effective than a physician-led model of care [4]. All the above reflects the higher level of confidence and appropriateness of physical therapists in the management of MSD than physicians [25].

**Figure 1:**
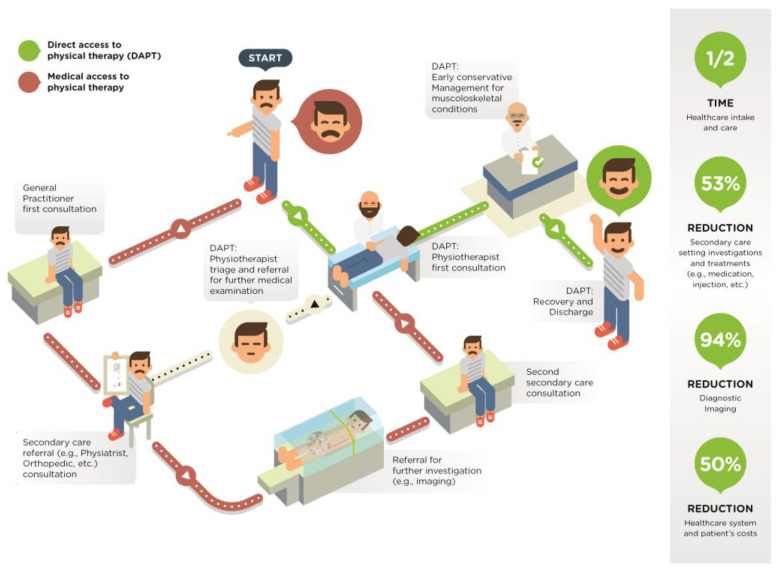
The main differences between access settings and the benefits offered by DAPT.

Regardless of the potential benefit, some countries still do not adopt DAPT; as an example, in Italy, it is still the general practice, especially in a public health setting (e.g., hospital), to adopt a physician-centered paradigm of care, where orthopedic or physiatrist physicians are the first point of contact for patients with simple to complex MSDs, even if physical therapy became an autonomous profession as early as 1994 [26]. Physician-centered care, cultural backgrounds, resistance to both changes and evidence-based practice may represent the main barriers preventing policymakers from adopting virtuous models, such as DAPT [27,28] Figure 1 illustrates the main differences between access settings and the benefits offered by DAPT.

Italian Law 42/1999 established the definitive overcoming of the auxiliary nature of the health professions, intellectually placing them at the same level of the medical profession, further defining the criteria for identifying their areas of autonomy and responsibility. Italian Law 251/2000 reaffirmed the health professions’ autonomy and responsibility; additionally, it recognized the core competence of functional diagnosis for the health professions of the rehabilitative area. Furthermore, Italian Law 43/2006 identified as “specialist” those health professionals who possess a postgraduate academic degree (in Italy, namely master). Recently, the Italian government has developed other legislative changes, such as Italian Law 24/2017 (namely, “Legge Gelli-Bianco”) [29] and Italian Law 3/2018 (namely, “Legge Lorenzin”) [30] that will profoundly affect the future professional practice. The first has modified, improved, integrated, and implemented the direct responsibility linked to the healthcare practice. At the same time, the second gave birth, for the first time after 60 years of “struggles”, to the Register of Physiotherapists inside the Italian Health and Care Professions Council (in Italy, called “Federazione Nazionale Ordini dei Tecnici Sanitari di Radiologia Medica e delle Professioni Sanitarie Tecniche, della Riabilitazione e della prevenzione”). Moreover, the Italian physical therapists’ community is rapidly increasing its role, competence, demand, and offer of postgraduate programs.

For example, hundreds of physical therapists achieve an international musculoskeletal certification, acquiring advanced knowledge and skills every year in seven postgraduate academic programs following the International Federation of Orthopedic Manipulative Physical Therapy standards [31]. Interestingly, although Law 43/2006 established the impossibility of creating new healthcare professionals that overlap with existing ones [32], the following Law 3/2018 introduced the recognition of new healthcare professionals that share similar competencies and scope of practice of physical therapy within the rehabilitation fields [30]. Italian institutions justified these new overlapping professions with the attempt to adapt the local healthcare system to the international scenario (e.g., osteopathy in France, chiropractic in the U.S.); however, it is unclear to the authors why the recognition of new professionals did not correspond to an empowerment of the long-established and autonomous physical therapy profession [27]. Within this new contest, it is unclear why DAPT is still not fully recognized, considering the historical context, evidence, and attempt to modernize by local institutions.

Thus, it is clear that the Italian context needs a paradigm change: physical therapy should be the first point of contact for MDS patients, and extended scope physical therapy should take place as an innovative role in the physical therapy profession and the local healthcare system [33]. The COVID-19 pandemic strongly threatened the sustainability of healthcare systems worldwide due to the complexity of patients’ symptoms [34] and an incessant demand for care [35], leading, in some cases, almost to collapse [36]. This emergency pointed out a long-lasting concern that complex healthcare systems and general practitioners should be relieved from managing certain health conditions, such as MSD, which may find adequate support by healthcare professionals, mainly from physical therapists [25,37].

## 3. Conclusions

The future is now: although still preliminary (e.g., the Italian context lacks high-quality primary studies), the evidence and previous experiences in other western countries supporting DAPT are promising. Additionally, given the expected shortage of physicians in the coming years, it is time to rethink the role of physical therapists within the healthcare system [38]. Hard skills, academic paths, scientific evidence, and legislative support indicate that this paradigm shift can take place in Italy. For these reasons, updating the knowledge offered during the educational pathway in physical therapy (from the bachelor’s degree to the doctor of philosophy degree) may improve clinical and reasoning skills and professional responsibility. Moreover, this change can also lead the profession to a more respectful and authoritative framing within the healthcare system, thus legitimizing the official recognition of the DAPT. By doing so, the enhanced standards of physical therapy may provide the profession with a unique opportunity to promote as physician extenders with a neuromusculoskeletal specialty aimed to become the standard providers of conservative care. That is, preliminary evidence suggests that post-professional specialization is a mainstay for developing advanced clinical, decision making, and reasoning skills level [39,40,41].

In summary, our call to action is addressed to the Italian Government and policymakers, and all other countries worldwide that do not yet provide this care pathway in their healthcare system organizations. According to the recent strategic recognition of DAPT in other European countries [16], we contend that the Italian physical therapy community is ready to achieve better professional recognition and to become the leading professionals in the first-line management of MSD [42], thus providing a valuable reference for citizens and the healthcare system.

## Figures and Tables

**Table 1:** All countries belonging to World Physiotherapy offering direct access [7].

Permitted	Private Only	Public Only	Not Allowed	Not Reported
Afghanistan	Albania	Benin	Austria	Bahrain
Australia	Argentina	Bhutan	Bahamas	Croatia
Bangladesh	Bermuda	Cameroon	Barbados	Curacao
Brazil	Bolivia		Belgium	Haiti
Canada	Bosnia Herzegovina		Bulgaria	Iran
Congo (Democratic Republic)	Cambodia		Chile	Mauritius
Costa Rica	Colombia		Czech Republica	Pakistan
Ecuador	Cyprus		Germany	Puerto Rico
Eswatini	Denmark		Greece	Sudan
Ethiopia	Estonia		Hong Kong	Syria
Finland	Fiji		Ivory Coast	Tanzania
Georgia	France		Jamaica	Zambia
Ghana	Hungary		Japan	
Guyana	Iceland		Jordan	
India	Indonesia		Korea (Republic of)	
Mali	Ireland		Kuwait	
Nepal	Israel		Lebanon	
New Zealand	Italy		Liechtestein	
Niger	Kenya		Malaysia	
Nigeria	Kosovo		Panama	
Papua New Guinea	Latvia		Perù	
Senegal	Lithuania		Philippines	
Singapore	Luxembourg		Romania	
South Africa	Macau		St Lucia	
Sri Lanka	Madagascar		Suriname	
Sweden	Malawi		Taiwan	
Thailand	Malta		Turkey	
Uganda	Mexico		Venezuela	
United Kingdom	Mongolia			
United States	Montenegro			
Zimbabwe	Morocco			
	Myanmar			
	Namibia			
	Netherlands			
	Norway			
	Poland			
	Portugal			
	Rwanda			
	Saudi Arabia			
	Slovakia			
	Slovenia			
	Spain			
	Switzerland			
	Togo			
	Trinidad Tobago			
	Ukraine			
	United Arab Emirates			
	Uruguay			

## Data Availability

Not applicable.
